# miR‐181a negatively modulates synaptic plasticity in hippocampal cultures and its inhibition rescues memory deficits in a mouse model of Alzheimer’s disease

**DOI:** 10.1111/acel.13118

**Published:** 2020-02-22

**Authors:** Carlos J. Rodriguez‐Ortiz, Gilberto Aleph Prieto, Alessandra C. Martini, Stefania Forner, Laura Trujillo‐Estrada, Frank M. LaFerla, David Baglietto‐Vargas, Carl W. Cotman, Masashi Kitazawa

**Affiliations:** ^1^ Department of Medicine University of California Irvine California; ^2^ Institute for Memory Impairments and Neurological Disorders University of California Irvine California; ^3^ Departamento de Neurobiología Celular y Molecular Instituto de Neurobiología Universidad Nacional Autonoma de México Querétaro Mexico; ^4^ Department of Neurobiology and Behavior University of California Irvine CA USA

**Keywords:** AMPA receptor, amyloid‐beta oligomers, GluA2, long‐term potentiation, microRNAs, spatial memory, translin/trax

## Abstract

MicroRNAs play a pivotal role in rapid, dynamic, and spatiotemporal modulation of synaptic functions. Among them, recent emerging evidence highlights that microRNA‐181a (miR‐181a) is particularly abundant in hippocampal neurons and controls the expression of key plasticity‐related proteins at synapses. We have previously demonstrated that miR‐181a was upregulated in the hippocampus of a mouse model of Alzheimer's disease (AD) and correlated with reduced levels of plasticity‐related proteins. Here, we further investigated the underlying mechanisms by which miR‐181a negatively modulated synaptic plasticity and memory. In primary hippocampal cultures, we found that an activity‐dependent upregulation of the microRNA‐regulating protein, translin, correlated with reduction of miR‐181a upon chemical long‐term potentiation (cLTP), which induced upregulation of GluA2, a predicted target for miR‐181a, and other plasticity‐related proteins. Additionally, Aβ treatment inhibited cLTP‐dependent induction of translin and subsequent reduction of miR‐181a, and cotreatment with miR‐181a antagomir effectively reversed the effects elicited by Aβ but did not rescue translin levels, suggesting that the activity‐dependent upregulation of translin was upstream of miR‐181a. In mice, a learning episode markedly decreased miR‐181a in the hippocampus and raised the protein levels of GluA2. Lastly, we observed that inhibition of miR‐181a alleviated memory deficits and increased GluA2 and GluA1 levels, without restoring translin, in the 3xTg‐AD model. Taken together, our results indicate that miR‐181a is a major negative regulator of the cellular events that underlie synaptic plasticity and memory through AMPA receptors, and importantly, Aβ disrupts this process by suppressing translin and leads to synaptic dysfunction and memory impairments in AD.

## Graphical abstract

In the hippocampus, neuronal stimulation produces upregulation of translin, reduction of miR‐181a, and an increase in the protein levels of its target GluA2 leading to synaptic plasticity. This plasticity mechanism is impaired by amyloid‐beta (Aβ) toxic species.

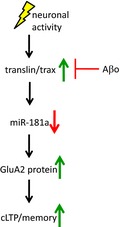

## 1 INTRODUCTION

Alzheimer's disease (AD) is the most common age‐related neurodegenerative disorder among elderly and is characterized by progressive deterioration of cognitive functions, particularly memory loss (Querfurth & LaFerla, 2010). Synapse dysfunction in the hippocampus and association cortices is an early pathological event that correlates best with the severity of cognitive decline. Therefore, an extensive number of studies has been conducted to better understand the underlying mechanisms by which neuropathological hallmarks, such as amyloid‐beta (Aβ) plaques, neurofibrillary tangles, and neuroinflammation, mediate synapse loss (Dickson et al., 1995; Sze et al., 1997; Terry et al., 1991; Walsh & Selkoe, 2004) and dysfunction (Prieto et al., 2017; Selkoe, 2002). In particular, glutamatergic synapses in the hippocampus are critically involved in memory formation and exhibit high plasticity to modify neuronal strength and connectivity. At the same time, they are vulnerable to various intrinsic and extrinsic insults (Stoltenburg‐Didinger, 1994; Walsh & Emerich, 1988). Therefore, unveiling the molecular mechanisms that perturb synaptic plasticity and promote synaptic degeneration in the hippocampus is indispensable to develop therapeutic interventions to halt the progression of cognitive deterioration in AD.

Synapses are highly dynamic, and their strength is rapidly adapted by changing local protein expression in an activity‐dependent manner (Eberwine, Miyashiro, Kacharmina, & Job, 2001; Job & Eberwine, 2001; Steward & Schuman, 2001). A growing number of reports has identified that microRNAs mediate such rapid and spatiotemporal modifications of local protein expression in synapses by degrading, halting, or stabilizing specific mRNAs without changing the transcriptomic profile of the entire cell (Costa‐Mattioli, Sossin, Klann, & Sonenberg, 2009; Dajas‐Bailador et al., 2012; Kosik, 2006; McNeill & Van Vactor, 2012; Muddashetty et al., 2011; Rajasethupathy et al., 2009; Schratt et al., 2006; Wang, Kwon, & Tsai, 2012). Among them, miR‐181a is an abundant microRNAs in the rodent hippocampus (Sambandan et al., 2017), which has recently been shown to modulate several plasticity‐related proteins including GluA2, CREB1, SIRT1, cFos, CaMKII, and PRKAA1 (Liu et al., 2013; Rodriguez‐Ortiz, Baglietto‐Vargas, Martinez‐Coria, LaFerla, & Kitazawa, 2014; Saba et al., 2012; Sambandan et al., 2017; Zhang et al., 2016; Zhang, Chen, Zhang, & Xu, 2017). Furthermore, we found that miR‐181a is significantly increased in the hippocampus of 3xTg‐AD mice in a pathology‐dependent manner and correlated with reduced levels of plasticity‐related proteins (Rodriguez‐Ortiz et al., 2014). In line with these findings, a recent study identified that plasma levels of miR‐181a were altered in MCI and early AD patients (Nagaraj et al., 2017). These reports suggest that miR‐181a plays a critical role in regulating activity‐dependent synaptic plasticity and memory formation, and its dysregulation may lead to accelerated loss of functional synapses and cognitive decline. Here, we investigated underlying molecular mechanisms by which neural stimulation modulated the levels of miR‐181a and the expression of its target plasticity‐related proteins in the absence or presence of Aβ species. We also determined whether inhibition of miR‐181a effectively alleviated cognitive deficits and positively modulated synaptic function in the 3xTg‐AD mouse model.

## 2 RESULTS

### 2.1 Learning promotes miR‐181a downregulation and increases GluA2 protein levels in the hippocampus

**Figure 1:**
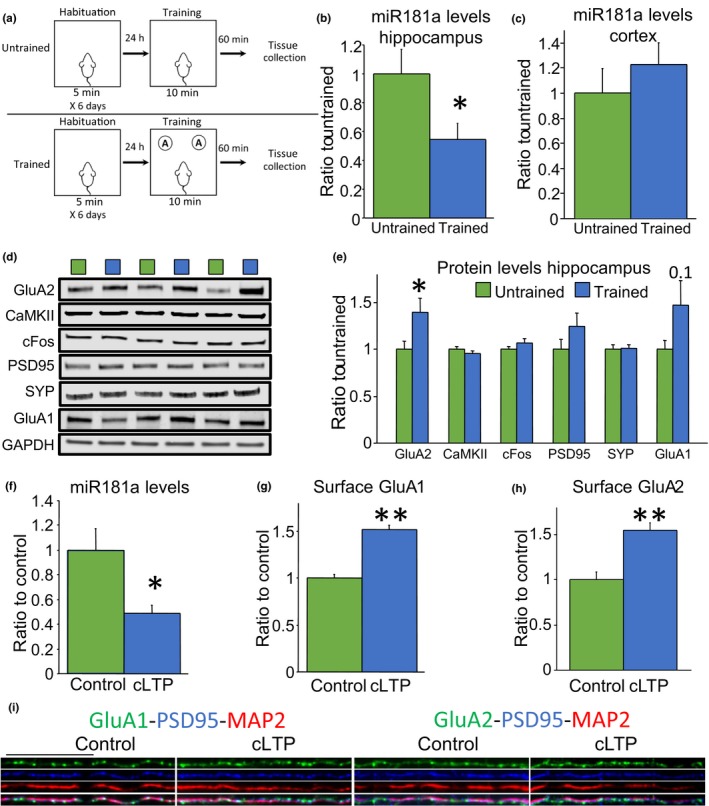
Hippocampal neuronal stimulation induced downregulation of miR‐181a and upregulation of GluA2 in vivo and in cultures. (a) Schematic representation of the learning episode used in these experiments (b) Hippocampal mature miR‐181a levels quantified by Taqman qPCR. microRNA levels were normalized to the small nuclear RNA U6 [*t*(_22_) = 2.26 *p* = .03, *n* = 12 per group]. (c) Cortical mature miR‐181a levels quantified by Taqman qPCR. microRNA levels were normalized to the small nuclear RNA U6 [*t*(_14_) = 0.87 *p* = .39, *n* = 7 and 9]. (d) Representative immunoblots of the data quantified in E. (e) Protein levels of the miR‐181a targets GluA2 [*t*(_24_) = 2.20 *p* = .03], CaMKII [*t*(_24_) = 0.97 *p* = .33], and cFos [*t*(_24_) = 1.24 *p* = .22]. Also are shown protein levels of the synaptic proteins PSD95 [*t*(_24_) = 1.39 *p* = .17], synaptophysin (SYP) [*t*(_23_) = 0.18 *p* = .85], and the AMPA receptor subunit GluA1 [*t*(_24_) = 1.68 *p* = .1]. Blots were normalized to GAPDH protein levels. *n* = 12–13 per group. * = *p* < .05 versus untrained. (f) Mature miR‐181a levels quantified by Taqman qPCR. microRNA levels were normalized to the small nuclear RNA U6 [*t*(_12_) = 2.71 *p* = .01, *n* = 7 per group]. * = *p* < .05 versus control. (g) Surface GluA1 quantification on dendrites [[*t*(_34_) = 8.26 *p* < .0001, *n* = 18 per group from three independent experiments]. *** = *p* < .0001 versus control. (h) Surface GluA2 quantification on dendrites [[*t*(_58_)=4.85 *p* < .001, *n* = 30 per group from three independent experiments].** = *p* < .001 versus control. (i) Representative immunofluorescence staining of surface GluA1 and GluA2 (green) on dendrites (labeled in red with microtubule associated protein 2, MAP2, and in blue with postsynaptic density protein 95, PSD95). For whole cell representative images, refer to Figure S1. Scale bar = 25µm. cLTP = chemical long‐term potentiation

First, we investigated whether miR‐181a is regulated in mice upon a learning episode. We trained 6‐month‐old wild‐type C57BL6/J mice in a well‐established object memory task (Vogel‐Ciernia & Wood, 2014). Animals were exposed to two identical objects for 10 min, and one hour later, hippocampal and cortical tissue were collected and analyzed for miR‐181a and its target plasticity‐related proteins (Figure 1a–e). We observed significantly reduced levels of miR‐181a in the hippocampus, but not the cortex, of trained animals (Figure 1b–c). In addition, we found augmented protein levels of the miR‐181a target GluA2 in the hippocampus after training (Figure 1d–e). No changes were observed in the protein levels of CaMKII and cFos, other two miR‐181a targets, in hippocampal samples of trained animals (Figure 1d–e). Similarly, no differences were observed in the synaptic proteins PSD95, synaptophysin (SYP), or the AMPA (α‐amino‐3‐hydroxy‐5‐methyl‐4‐isoxazolepropionic acid) receptors subunit GluA1 at this time point (Figure 1d–e). These results show learning‐induced downregulation of miR‐181a that correlates with augmented protein levels of its target GluA2 in the hippocampus.

### 2.2 Neuronal activity reduces miR‐181a levels in primary hippocampal cultures

In addition, we investigated whether miR‐181a downregulation occurs by neuronal stimulation in hippocampal cultures. Sambandan and colleagues have recently demonstrated that electrical stimulation triggers miR‐181a processing at synapses (Sambandan et al., 2017). Here, we induced chemical long‐term potentiation (cLTP) by incubating primary hippocampal cultures with 0.02 mM glycine for ten minutes. One hour after cLTP, we found a significant reduction of miR‐181a in the cultures (Figure 1f). In parallel, we detected increased levels of surface GluA1 and GluA2, reflecting the incorporation of AMPA receptors to the membrane (Figure 1g‐I), an essential event for LTP (Park et al., 2006; Shi et al., 1999).

### 2.3 miR‐181a mimics disrupt plasticity in hippocampal cultures

**Figure 2:**
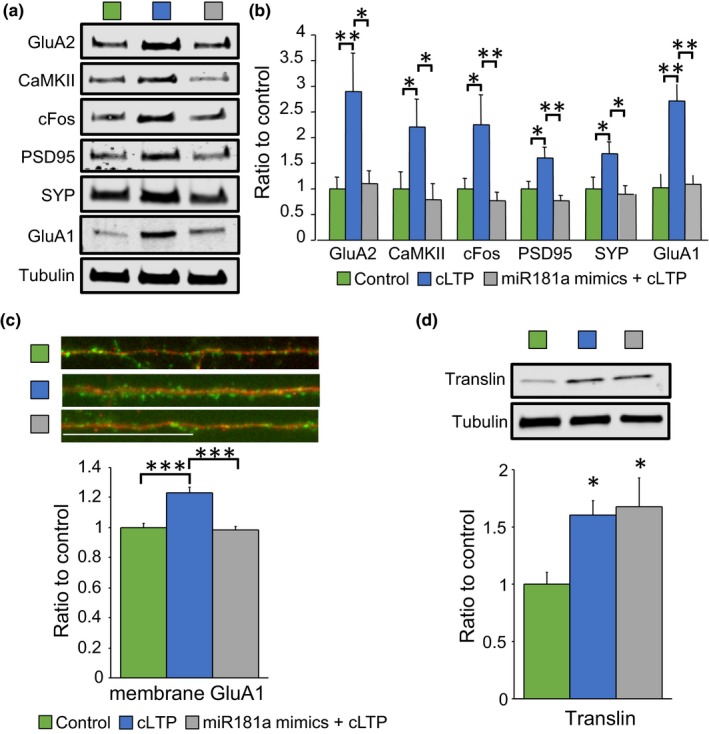
cLTP‐dependent induction of plasticity‐related proteins was prevented by miR‐181a mimics. (a) Representative immunoblots of the data quantified in B. (b) Protein levels of the miR‐181a targets GluA2 [ANOVA (*F*(_2,27_)=4.93 *p* = .01, *n* = 10], CaMKII [ANOVA (*F*(_2,30_)=3.59 *p* = .04, *n* = 11], and cFos [ANOVA (*F*(_2,27_)=4.87 *p* = .01, *n* = 10]. Also are shown protein levels of the synaptic proteins PSD95 [ANOVA (*F*(_2,27_)=6.95 *p* = .003, *n* = 10], synaptophysin (SYP) [ANOVA (*F*(_2,30_)=4.31 *p* = .02, *n* = 11], and the AMPA receptor subunit GluA1 [ANOVA (*F*(_2,15_)=13.65 *p* = .0004, *n* = 6]. Blots were normalized to tubulin protein levels. * = *p* < .05, ** = *p* < .01. (c) Immunofluorescence of surface GluA1 levels (green) on dendrites (labeled in red with microtubule associated protein 2, MAP2) [ANOVA (*F*(_2,87_)=21.44 *p* < .0001, *n* = 30 per group from three independent experiments)]. *** = *p* < .0001. Scale bar = 25µm. cLTP = chemical long‐term potentiation. (d) Protein levels of the RNA‐binding protein translin [ANOVA (*F*(_2,15_)=4.54 *p* = .02, *n* = 6 per group]. Blots were normalized to tubulin protein levels. * = *p* < .05

Next, we evaluated whether miR‐181a target proteins entailed in synaptic plasticity were modulated by cLTP in hippocampal cultures. Figure 2a–b shows that the protein levels of the miR‐181a targets, GluA2, CaMKII, and cFos, were significantly increased one hour after stimulation (blue bars). Furthermore, we found that PSD95, SYP, and GluA1 were also augmented following cLTP (Figure 2a–b, blue bars). These activity‐dependent changes were significantly mitigated by cotreatment with synthetic miR‐181a in primary cultures (Figure 2a–b, gray bars). Recruitment of GluA1 to the cell surface following cLTP was also significantly impaired in the presence of synthetic miR‐181a mimics (Figure 2c), indicating that miR‐181a contributes directly to activity‐induced plasticity.

To explore in further detail the mechanisms that lead to miR‐181a‐dependent plasticity, we analyzed translin protein levels, a brain‐enriched RNA‐binding protein proven to translocate to dendrites in response to neuronal stimulation and involved in microRNA degradation (Asada et al., 2014; Han, Gu, & Hecht, 1995a; Han, Yiu, & Hecht, 1995b; Park et al., 2017; Wu et al., 2011). We observed increased translin protein levels in hippocampal cultures after cLTP and, in contrast to the other proteins analyzed, translin levels were not affected by synthetic miR‐181a mimics (Figure 2d). These results are consistent with the idea that translin is involved in activity‐induced plasticity upstream miR‐181a.

### 2.4 Aβo‐induced deficits in plasticity are rescued by miR‐181a inhibition in primary hippocampal cultures

**Figure 3:**
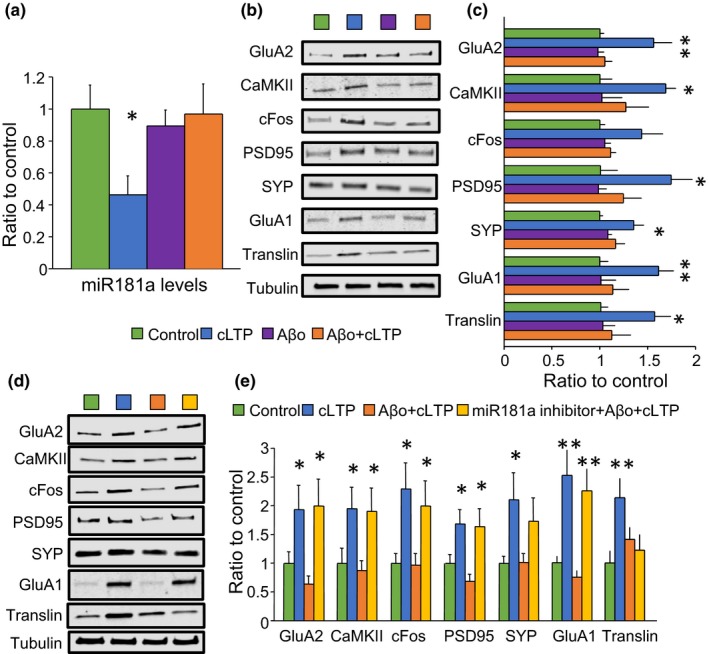
miR‐181a inhibition restored Aβo‐induced impairments in plasticity‐related proteins. (a) Mature miR‐181a levels quantified by Taqman qPCR. microRNA levels were normalized to the small nuclear RNA U6 [ANOVA (*F*(_3,19_)=3.19 *p* = .03, *n* = 9 per group]. * = *p* < .05. (b) Representative immunoblots of the data quantified in C. (c) Protein levels of the miR‐181a targets GluA2 [ANOVA (*F*(_3,16_)=6.26 *p* = .005], CaMKII [ANOVA (*F*(_3,15_)=3.56 *p* = .03], and cFos [ANOVA (*F*(_3,16_)=2.95 *p* = .06)]. Also are shown protein levels of the synaptic proteins PSD95 [ANOVA (*F*(_3,12_)=4.01 *p* = .03], synaptophysin (SYP) [ANOVA (*F*(_3,16_)=4.25 *p* = .02], the AMPA receptor subunit GluA1 [ANOVA (*F*(_3,16_)=3.99 *p* = .02], and the RNA‐binding protein translin [ANOVA (*F*(_3,16_)=3.23 *p* = .05]. Blots were normalized to tubulin protein levels. *n* = 4–5 per group. (d) Representative immunoblots of the data quantified in E. (e) Protein levels of the miR‐181a targets GluA2 [ANOVA (*F*(_3,40_)=4.02 *p* = .01, *n* = 11], CaMKII [ANOVA (*F*(_3,44_)=3.31 *p* = .02, *n* = 12], and cFos [ANOVA (*F*(_3,44_)=3.95 *p* = .01, *n* = 12]. Also are shown protein levels of the synaptic proteins PSD95 [ANOVA (*F*(_3,40_)=4.75 *p* = .006, *n* = 11], synaptophysin (SYP) [ANOVA (*F*(_3,44_)=2.85 *p* = .04, *n* = 12], the AMPA receptor subunit GluA1 [ANOVA (*F*(_3,20_)=8.61 *p* = .0007, *n* = 6], and the RNA‐binding protein translin [ANOVA (*F*(_3,19_)=3.78 *p* = .02, *n* = 6]. Blots were normalized to tubulin protein levels. * = *p* < .05, ** = *p* < .01 versus control. cLTP = chemical long‐term potentiation; Aβo = amyloid‐beta oligomers

We have previously reported a significant elevation of miR‐181a in the hippocampus of 3xTg‐AD mice in pathology‐ and age‐dependent manners (Rodriguez‐Ortiz et al., 2014). Robust evidence supports that Aβ oligomers (Aβo) disrupt LTP (Baglietto‐Vargas et al., 2018; Lambert et al., 1998; Walsh et al., 2002), reviewed in Viola and Klein (2015). Thus, we treated primary hippocampal cultures with 100 nM Aβo for two hours and analyzed miR‐181a levels and plasticity‐related proteins one hour after cLTP stimulation. We found that Aβo treatment prior to cLTP effectively prevented downregulation of miR‐181a (Figure 3a). Moreover, Aβo treatment significantly impaired upregulation of activity‐induced proteins, including GluA2, CaMKII, cFos, PSD95, SYP, GluA1, and translin (Figure 3b–c, orange bars). To determine whether miR‐181a mediates Aβo‐dependent deficits in plasticity, we inhibited miR‐181a in cultures treated with Aβo and subjected to cLTP. We observed a significant increase in the protein levels of GluA2, CaMKII, cFos, PSD95, and GluA1 upon cLTP despite Aβo treatment when miR‐181a was inhibited (Figure 3d–e, yellow bars). Importantly, translin protein levels were not rescued by miR‐181a inhibition, consistent again with translin being upstream to miR‐181a in the molecular cascade triggered by neuronal stimulation (Figure 3d–e, yellow bars). These results support the view that reduction of miR‐181a is an important activity‐dependent event that can be dysregulated by Aβ toxic species and that inhibition of miR‐181a suffices to counteract the detrimental consequences of Aβo in culture.

### 2.5 miR‐181a and plasticity‐induced protein levels are dysregulated in hippocampal crude synaptosome preparations of 3xTg‐AD animals

**Figure 4:**
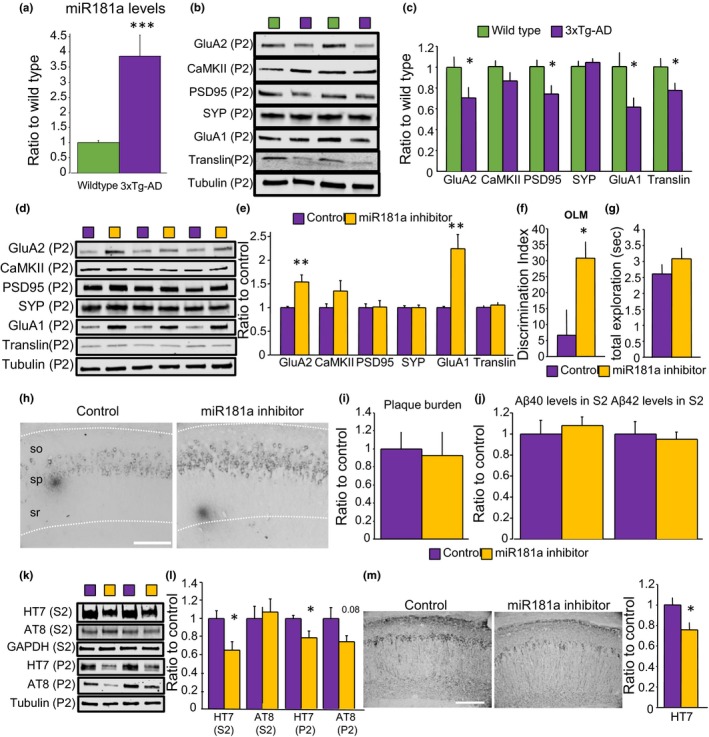
miR‐181a inhibition in the hippocampus of 3xTg‐AD animals increased synaptic GluA1 and GluA2 protein levels, rescued object location memory impairments, and diminished tau protein levels. (a) Mature miR‐181a levels quantified by Taqman qPCR in hippocampal P2 fractions of 13‐month‐old wild‐type and 3xTg‐AD mice. microRNA levels were normalized to 18S ribosomal RNA [*t*(_37_) = 4.06 *p* = .0002, *n* = 20 and 19]. (b) Representative immunoblots of the data quantified in C. (c) Protein levels in the P2 fractions of the hippocampus of 13‐month‐old wild‐type and 3xTg‐AD mice. GluA2 [*t*(_25_) = 2.11 *p* = .04], CaMKII [*t*(_27_) = 1.43 *p* = .16], PSD95 [*t*(_28_) = 2.53 *p* = .01], synaptophysin (SYP) [*t*(_28_) = 0.53 *p* = .59], GluA1 [*t*(_27_) = 2.36 *p* = .02], and translin [*t*(_27_) = 2.13 *p* = .04]. Blots were normalized to tubulin protein levels. *n* = 13–16 per group. * = *p* < .05 versus wild‐type. (d) Representative immunoblots of the data quantified in E. (e) Protein levels in the P2 fractions of the hippocampus of 13‐month‐old injected 3xTg‐AD mice. GluA2 [*t*(_21_) = 3.61 *p* = .001], CaMKII [*t*(_21_) = 1.37 *p* = .18], PSD95 [*t*(_21_) = 0.14 *p* = .88], synaptophysin (SYP) [*t*(_20_) = 0.14 *p* = .88], GluA1 [*t*(_21_) = 4.13 *p* = .0005], and translin [*t*(_18_) = 0.77 *p* = .45]. Blots were normalized to tubulin protein levels. *n* = 9–12 per group. ** = *p* < .01 versus control. (f) Object location memory (OLM) discrimination index in 13‐month‐old 3xTg‐AD mice injected with AAV control or AAV miR‐181a inhibitor [*t*(_38_) = 2.60 *p* = .013, *n* = 19 and 21]. * = *p* < .05 versus control. (g) Total exploration time in seconds (sec) on the test [*t*(_38_) = 1.06 *p* = .29]. (h) Representative image of the staining with 6E10 for a control (left) and a mouse injected with miR‐181a inhibitor (right). so = stratum oriens, sp = stratum pyramidale, sr = stratum radiatum. Scale bar = 100µm. (i) Analysis of Aβ plaques [*t*(_8_) = 0.21 *p* = .83, *n* = 4 and 6]. (j) Measurements by electrochemiluminescence sandwich immunoassay of Aβ40 [*t*(_33_) = 0.50 *p* = .61] and Aβ42 [*t*(_33_) = 0.37 *p* = .70] in the S2 fraction of the hippocampus of injected mice. *n* = 17 and 18. (k) Representative immunoblots of the data quantified in L. (l) Protein levels of total tau (measured by the HT7 antibody, normalized to GAPDH) [*t*(_30_) = 2.69 *p* = .011, *n* = 15 and 17], and phosphorylated paired helical filament tau at residues Ser202/Thr205 (measured by the AT8 antibody, normalized to HT7) [*t*(_28_) = 0.37 *p* = .71, *n* = 15] in the S2 and P2 fractions of the hippocampus of injected mice. * = *p* < .05. (m) Analysis of HT7 staining [*t*(_8_) = 2.42 *p* = .04, *n* = 4 and 6] and representative images. Scale bar = 100µm. P2 = crude synaptosome fraction, S2 = soluble fraction

We then investigated the possible role of miR‐181a in plasticity‐related events in a mouse model of AD. For this, we first prepared crude synaptosome fractions from hippocampus homogenates of 13‐month‐old wild‐type and 3xTg‐AD mice. Our synaptosome preparations showed significantly larger levels of the synaptic protein PSD95 and low contamination with nuclear, astrocytes, and microglia markers, indicating that we obtained a synapse‐enriched fraction (Figure S3). Consistent with our previous studies (Rodriguez‐Ortiz et al., 2014), we observed increased levels of miR‐181a in the hippocampal synaptosome fraction (P2) of 3xTg‐AD compared to wild‐type mice (Figure 4a). Furthermore, the protein levels of GluA2, PSD95, GluA1, and translin were significantly lower in hippocampal synaptosome samples from 3xTg‐AD animals (Figure 4b–c). These observations are in agreement with other reports showing synaptic impairments in the 3xTg‐AD model (Baglietto‐Vargas et al., 2018; Caccamo, Maldonado, Bokov, Majumder, & Oddo, 2010; Clark et al., 2015; Oddo et al., 2003).

### 2.6 Augmented protein levels of the AMPA receptor subunits GluA2 and GluA1 are observed in hippocampal crude synaptosome preparations of 3xTg‐AD mice after miR‐181a inhibition

Then, we examine the molecular mechanisms elicited by miR‐181a inhibition in synapses of 3xTg‐AD animals. For this, we infused viral particles containing a double‐strand RNA sequence against miR‐181a or a no related control sequence into the hippocampus of 10‐month‐old 3xTg‐AD mice. The particles also included the GFP gene to easily monitor expression. Three months after injection in the hippocampus, effective GFP protein expression was observed by immunoblot in total homogenates, as well as in synaptosome‐enriched fractions by flow cytometry, thus confirming that viral particles targeted neurons (Figure S2). The protein levels of GluA2 and GluA1 were significantly higher in crude synaptosome samples collected from mice injected with the miR‐181a inhibitor compared to mice injected with control particles (Figure 4d–e). No differences were detected in the levels of CaMKII, PSD95, SYP, translin, or cFos between the treatments (Figure 4d–e) and Figure S4.

### 2.7 Object location memory deficits are rescued by miR‐181a inhibition in 3xTg‐AD mice

Infused 3xTg‐AD mice were trained in an object location memory (OLM) protocol at 13 months of age (Vogel‐Ciernia & Wood, 2014). We specifically used this test to evaluate hippocampus‐dependent spatial memory function of 3xTg‐AD mice because OLM test allows high sensitivity and accurate assessment of memory without being influenced by inherent stress, emotional distress, and other exogenous factors associated with the nature and procedure of most behavioral tests used in rodents (*e.g.,* swimming, shocks) (Vogel‐Ciernia & Wood, 2014). Figure 4f shows that 3xTg‐AD animals presented clear long‐term memory deficits that were rescued by the infusion of the miR‐181a inhibitor. No significant differences were unveiled in the total exploration time between the groups (Figure 4g). In all, our in vivo results are consistent with the idea that miR‐181a inhibition restored synapse function and cognition in 3xTg‐AD mice.

### 2.8 AD pathology is differentially affected by miR‐181a inhibition in 3xTg‐AD mice

Our data showed that inhibition of miR‐181a effectively reversed synaptic impairments of Aβo in culture, suggesting that miR‐181a dysregulation is downstream of Aβ pathology. To test whether miR‐181a can modulate Aβ pathology in vivo, we examined the hippocampus of injected 3xTg‐AD mice for Aβ plaques by immunohistochemistry and found no differences between miR‐181a inhibitor and control treatments (Figure 4h‐I). Moreover, the levels of soluble Aβ species, Aβ40 and Aβ42, in the hippocampus were similar between the treatments (Figure 4j). These results suggest that miR‐181a inhibition does not modify Aβ pathology in the hippocampus of 3xTg‐AD mice.

On the other hand, we detected a significant reduction of total tau levels in the soluble (S2) and crude synaptosome fractions (P2) of the hippocampus of 3xTg‐AD mice that received miR‐181a inhibitor (Figure 4k‐L). Phosphorylated tau at residues Ser202/Thr205 was unchanged in the soluble fraction but showed a trend to significance in crude synaptosomes of the miR‐181a inhibitor group (Figure 4k‐L). In addition, analysis of immunohistochemistry experiments for total tau revealed significant differences in the hippocampus of mice infused with miR‐181a inhibitor compared to control (Figure 4m).

## 3 DISCUSSION

Our present study highlights that miR‐181a is a negative regulator for synaptic plasticity and mediates Aβ‐induced synaptotoxicity. cLTP in cultures and cognitive stimulation in vivo reduce the levels of miR‐181a and upregulate GluA2, in which mRNA is a proven target for miR‐181a. Inhibition of miR‐181a effectively reverses Aβo‐induced impairments in plasticity and memory deficits in primary hippocampal cultures and 3xTg‐AD mice through restoration of GluA2 and GluA1 protein levels in the hippocampus.

miR‐181a is abundantly expressed in cultured neurons of the hippocampus, and its maturation is rapidly triggered by electrical stimulation to modulate CaMKII in dendrites (Sambandan et al., 2017). In addition, miR‐181a can inhibit protein expression of CREB1 (Liu et al., 2013), a transcription factor that plays a well‐known role in learning and memory (Gao et al., 2010). Direct regulation of CREB1 by miR‐181a was tested in HEK293 cells using a luciferase reporter containing the 3′‐UTR of the CREB1 mRNA. The authors detected diminished luciferase activity when the cells were cotransfected with miR‐181a mimics. In rat primary neurons, transfection with miR‐181a mimics caused significant reduction in dendritic length and branching, and the opposite results were observed with transfection of a miR‐181a inhibitor (Liu et al., 2013). Furthermore, an in vivo study found that miR‐181a levels, together with other 3 microRNAs, were upregulated in the hippocampus of epileptic rats that presented memory deficits but not in the animals unimpaired by the epileptic treatment (Liu et al., 2015). The same group later reported that overexpression of miR‐181a in the brain by injections of a miR‐181a agonist in the lateral ventricle resulted in significant memory impairment (Huang et al., 2015). Conversely, a recent report showed that training in contextual fear conditioning and object location induced upregulation of miR‐181a in the hippocampus, which resulted in inhibition of 5' AMP‐activated protein kinase (AMPK) activity by reducing the levels of its catalytic subunit PRKAA1. Consistently, miR‐181a inhibition impaired memory and augmented PRKAA1 protein expression, suggesting that miR‐181a is a positive regulator of memory (Zhang et al., 2017). These apparently opposite findings, including our present study, strongly implicate that miR‐181a plays a pivotal role in regulating plasticity and synapse functions, although further research is essential to determine the specific mechanisms by which miR‐181a controls plasticity in various spatiotemporal conditions.

Despite many microRNAs are localized at the dendritic compartment and are found to be involved in synaptic plasticity and memory (Griggs, Young, Rumbaugh, & Miller, 2013; Zhang et al., 2017), little is known about the mechanisms linking synaptic stimulation and regulation of microRNAs. The protein complex composed of the RNA‐binding protein translin and the RNase trax (translin‐associated factor X) has been shown to be enriched in the brain and recruited to dendrites upon neuronal activity (Han, Gu, et al., 1995a; Han, Yiu, et al., 1995b; Wu et al., 2011). More recently, it has been reported that translin/trax degrades microRNAs and therefore alleviates translational repression (Asada et al., 2014; Park et al., 2017). Here, we found downregulation of miR‐181a and upregulation of translin upon cLTP in hippocampal primary cultures. cLTP‐dependent modulation of both, miR‐181a and translin, was dysregulated by Aβ oligomers. Interestingly, miR‐181a inhibition did not rescue Aβo‐induced translin deficits despite reinstating the levels of all the other proteins analyzed. Similar results were obtained in vivo where we observed dysregulation of miR‐181a and translin in the hippocampal synaptosome fraction of 3xTg‐AD mice and failure to restore translin protein levels by inhibiting miR‐181a in the hippocampus of the AD model. These findings support the hypothesis that the complex translin/trax is essential in activity‐induced plasticity upstream miR‐181a, mechanism that is altered in the 3xTg‐AD model.

Subunits of the AMPA receptors are subjected to tight regulation because of their pivotal roles in synaptic plasticity. Regulation includes phosphorylation and translocation to the membrane (Park et al., 2006; Shi et al., 1999), endocytosis (Awasthi et al., 2019), and local translation (Ju et al., 2004; Kacharmina, Job, Crino, & Eberwine, 2000). GluA2 is one of the main AMPA receptor subunits expressed in the hippocampus (Lu et al., 2009), and mRNAs of GluA1 and GluA2 are found in dendrites where they are synthesized locally and incorporated into the membrane at or near synapses (Ju et al., 2004). GluA2 regulation by miR‐181a has been explored in cultured cells where it was shown that overexpression of miR‐181a reduces GluA2, while miR‐181a knockdown reverses it, and loss of GluA2 leads to a reduction of spines formation and diminished basal synaptic transmission (Zhang et al., 2016). Our study is in line with these reports and supports that miR‐181a is fundamental to the expression of GluA2 in response to activity in cultures and in vivo. In addition to GluA2, we observed regulation of other plasticity‐related proteins upon cLTP but not after learning. These results may be related to the fact that cLTP involves continuous chemical activation of a large set of synapses that increases the probability of detecting molecular changes in the cells (MacDonald, Ju, & Wang, 2001; Molnar, 2011; Salter, 2001), while learning is a restricted stimulation that produces tightly controlled activation of a small set of synapses within the network, and detection of these synaptic modifications may be challenging when most of the synapses did not change (Whitlock, Heynen, Shuler, & Bear, 2006).

Our study revealed that reduction of miR‐181a in 3xTg‐AD mice decreased soluble and synaptosome‐enriched tau in the hippocampus. It has previously been reported that tau accumulation in synapses favors phosphorylation of the GluN2B subunit of NMDA receptors leading to association with PSD95 and excitotoxicity (Ittner et al., 2010). These results suggest that, in addition to modulation of plasticity‐related targets, miR‐181a dysregulation may indirectly contribute to synaptic and memory impairments by modifying neuronal tau levels. Recent evidence indicates that the mammalian target of rapamycin (mTOR) pathway may be the connection between miR‐181a and tau. As mentioned above, miR‐181a can negatively control the expression of PRKAA1 and therefore reduce AMPK activity (Zhang et al., 2017). AMPK is a negative regulator of mTOR signaling that in turn positively modulates total and phosphorylated tau levels in the brain of neurodegenerative mouse models (Caccamo et al., 2013).

Our results showed that miR‐181a inhibition alleviated to some extend synapse dysfunction and impaired cognition in 3xTg‐AD animals. In addition, we found that Aβ oligomers disrupted synaptic plasticity by dysregulating the levels of miR‐181a. These results strengthen the idea that synapse degeneration is mediated by microRNAs in AD and highlights the importance of studying microRNAs that control synaptic plasticity and their underlying mechanisms in the search of AD molecular markers and therapeutic targets.

## 4 EXPERIMENTAL PROCEDURES

### 4.1 Primary culture

Hippocampal primary cells were collected from postnatal day 0 C57BL/6J mice. Cells were grown and fed twice a week with Neurobasal medium with antibiotics and supplemented with GlutaMAX and B‐27 (Thermo Fisher Scientific). Chemical long‐term potentiation (cLTP) was induced in 14‐ to 16‐day‐old cells using a modified protocol from Park et al. (2006). First, cells were washed for 5 min with extracellular solution containing [mM]: 120 NaCI, 3 KCl, 2 CaCl2, 2 MgCl2 15 glucose, 15 HEPES, and pH 7.4. For glycine stimulation, cells were treated for 10 min with 0.2 mM glycine in a solution containing [mM]: 150 NaCI, 5 KCl, 2 CaCl2, 30 glucose, 10 HEPES, 0.001 strychnine, 0.02 bicuculline, and pH 7.4. Cells were returned to extracellular solution and incubated for an additional 50 min before lysis or fixation.

For immunoblot analysis, cells were washed with ice‐cold PBS. Then, M‐PER complemented with proteases and phosphatases inhibitors (Thermo Fisher Scientific) was added, and cells were scrapped and centrifuged at 12,000 × *g* for 10 min at 4°C. Protein concentration in the supernatant was determined using a commercial Bradford assay (Bio‐Rad).

In the experiments described in Figures 2 and 3, cultures were transfected 48h before cLTP with 20nM of one of the followings: hsa‐miR‐181a‐5p mirVana miRNA mimic (ID MC10421), mirVana miRNA mimic negative control #1 (cat. 4,464,058), hsa‐miR‐181a‐5p mirVana miRNA inhibitor (ID MH10421), or mirVana miRNA inhibitor negative control #1 (cat. 4,464,076) (all from Thermo Fisher Scientific) using Lipofectamine RNAiMAX (Thermo Fisher Scientific) following the manufacturer's protocol.

In the experiments described in Figure 3, cells were treated for 2h with 100nM amyloid‐beta‐derived diffusible ligands 1‐42, ADDLs (Aβ oligomers) before cLTP induction. Aβ oligomers were prepared according to the protocol previously reported by Klein (2002). Aβ oligomers concentration was determined by NanoDrop (Thermo Fisher Scientific) immediately before applying to the cells.

### 4.2 Mice and surgery

Six‐month‐old male wild‐type C57BL/6J mice were used in the experiments presented in Figure 1. Experiments presented in Figure 4 were conducted in male homozygous 3xTg‐AD mice or their respective genetic control (hybrid 129/C57BL6). Bilateral stereotaxic infusions into the hippocampus were performed in 10‐month‐old mice under continuous isoflurane anesthesia. A 2 µl of adeno‐associated viral particles AAV9‐GFP‐U6‐mmu‐Mir181a‐5p‐shRNA or AAV9‐GFP‐U6‐scramble‐shRNA (~10^13^ gc/ml determined by qPCR; Vector Biolabs) was infused over 2 min using a 10‐μl Hamilton microsyringe driven by a microinfusion pump in the following coordinates (from Bregma): posterior 2.06 mm, lateral ± 1.75 mm, and ventral 1.95 mm (Franklin & Paxinos, 2007). The injection needle was left in place for an additional 2 min to allow complete diffusion of the particles. After recovery on a heating pad, animals were returned to their home cages. Behavioral experiments started 3 months after infusions when the mice were 13 months of age.

Mice were housed 2–5 per cage and maintained at ~21°C under a 12 light/12 dark cycle with food and water freely available. All protocols were carried out during the light phase. All animal procedures were performed in accordance with National Institutes of Health and University of California guidelines and were approved by the Institutional Animal Care and Use Committee at the University of California, Irvine.

### 4.3 Object location memory

Mice were handled for three days before starting habituation to the arena. Each mouse was habituated for 6 consecutive days to a white acrylic 5‐sided box (30.5 cm × 30.5 cm × 30.5 cm) with the illumination set at ~48 LUX. A black vertical stripe was fixed on one of the walls of the arena, and the floor was covered with Sani‐Chips bedding (P.J. Murphy Forest Products). On training (day 7), mice were exposed to two identical objects (100‐ml beakers filled with cement) placed at opposite ends of the arena for 10 min. On test (24 hr later, day 8), one of the objects was moved to a novel location within the arena and mice were allowed to explore for 5 min. Objects and their relative positions were counterbalanced, and, to avoid olfactory cues, objects were thoroughly cleaned with 70% ethanol, and bedding was stirred after each trial. A video camera was mounted above the arena, and all sessions were recorded. Videos were hand‐scored offline, and discrimination index was calculated using the formula: time exploring the novel object – time exploring the familiar)/(total exploration time) × 100. Exploration was considered as pointing the head toward an object. Turning around, chewing or sitting on the objects was not considered as exploratory behavior.

In the experiments described in Figure 1, animals were handled and habituated as above. On training, one group had an extra habituation session of 10 min (untrained) and the other was exposed to two identical objects as described above (trained). All animals were euthanatized one hour after this session (day 7), and the hippocampus and cortex were dissected and frozen in dry ice (Figure 1a).

### 4.4 Tissue preparation

After deep anesthesia with carbon dioxide, the brain was quickly collected, washed with ice‐cold PBS, and dissected. For immunohistochemistry analysis, half brain was fixed 48 hr in PBS + 4% paraformaldehyde and embedded in paraffin. Whole hippocampal and cortical lysates were prepared by homogenizing frozen tissue in T‐Per extraction buffer (150 mg/ml, Pierce), complemented with proteases and phosphatases inhibitors (Fisher Scientific). Lysates were centrifuged at 20,000 × *g* for 30 min at 4ºC.

For crude synaptosomes, the hippocampus was dissected, placed in 1.5 ml 320 mM sucrose + 10 mM HEPES (pH 7.4) complemented proteases and phosphatases inhibitors (Fisher Scientific), and frozen at a controlled rate (−1°C/min) in CoolCell containers (Corning) in a −80°C freezer until further processing. Tissue was homogenized by hand using a glass–teflon grinder. Homogenates were centrifuged at 1,200×*g* for 10 min. The supernatant was further centrifuged at 12,500 × g for 20 min. The resulting pellet was resuspended in T‐Per complemented with proteases and phosphatases inhibitors and centrifuged at 1,200xg for 10 min (Figure S3a). All centrifugations were carried out at 4ºC. Protein concentrations were determined using a commercial Bradford assay (Bio‐Rad).

### 4.5 Immunoblotting

Equal amounts of protein were separated on 4%–15% Bis‐Tris gel and transferred to PVDF membranes. Membranes were blocked for 1 hr in Odyssey blocking solution (Li‐cor). After blocking, membranes were incubated overnight with one or two primary antibodies (Table 1) in Odyssey blocking solution + 0.2% Tween‐20 at 4°C. After washes with TBS + 0.1% Tween‐20, membranes were incubated for 2 hr with the specific secondary antibody(ies) (IRDye, Li‐cor) at a 1:20,000 dilution in TBS + 5% nonfat Milk + 0.2% Tween‐20 + 0.01% SDS. Blots were scanned in an Odyssey infrared imager (Li‐cor), and the Image Studio software v5.2 (Li‐cor) was used for quantification.

**Table 1:** List of primary antibodies used in this study

Target	Vendor and catalog number	Application and dilution
GluA1	Cell Signaling, 13,185	IF (1:400), WB (1:1,000)
Map2	Abcam, ab5392	IF (1:500)
GluA2	MilliporeSigma, MAB397	IF (1:200), WB (1:1,000)
CaMKII	Cell Signaling, 3,362	WB (1:1,000)
cFos	Abnova. PAB14840	WB (1:1,000)
PSD95	Cell Signaling, 3,409	IF (1:100), WB (1:1,000)
PSD95	NeuroMab, 73‐028	IF (1:100)
SYP	Abcam, ab14692	WB (1:2000)
tubulin	Sigma‐Aldrich, B‐5‐1‐2	WB (1:25,000)
GFP	Cell Signaling, 2,955	WB (1:1,000)
GAPDH	ProteinTech, 60004‐1‐Ig	WB (1:5,000)
H3	Cell Signaling, 4,499	WB (1:1,000)
GFAP	Dako, Z0334	WB (1:3,000)
CD68	Biorad, MCA1957	WB (1:200)
HT7	Thermo Fisher Scientific, MN1000	WB (1:1,000), IHC (1:500)
AT8	Thermo Fisher Scientific, MN1020	WB (1:1,000)
6E10	BioLegend, SIG−39320	IHC (1:1,000)
translin	Abcam, ab166827	WB (1:1,000)

Abbreviations: WB, Western blot; IF, immunofluorescence; IHC, immunohistochemistry

### 4.6 RNA isolation, reverse transcription, and real‐time polymerase chain reaction

Total RNA was isolated from primary cells using Direct‐zol RNA MicroPrep (Zymo Research Corp). Total RNA from tissue lysates was obtained using Direct‐zol RNA Miniprep (Zymo Research Corp). RNA concentrations were determined using a NanoDrop spectrophotometer (Thermo Fisher Scientific). cDNA was produced from 10ng RNA using TaqMan MicroRNA Reverse Transcription Kit or from 50ng RNA using High Capacity cDNA Reverse Transcription Kit (18S assays) (Thermo Fisher Scientific). Quantitative RT‐PCR was performed using a CFX Connect Real‐Time System (Bio‐Rad) with the TaqMan assays hsa‐miR‐181a (ID 000480), U6 snRNA (ID 001973), and mouse 18S (Rn18s Rn45s, ID Mm03928990_g1) (Thermo Fisher Scientific). Cycling for PCR amplification was as follows: enzyme activation at 95°C for 10 min, followed by 40 cycles at 95°C for 15 s and at 60°C for 60sec.

### 4.7 Immunofluorescence

After treatments, hippocampal cultures were washed with ice‐cold PBS and fixed in PBS + 4% paraformaldehyde (pH 7.4) for 10 min. After washes, cells were blocked in PBS 1% bovine serum albumin (BSA) 10% goat serum for 1 hr and incubated overnight at 4ºC with GluA1 or GluA2 primary antibody. Cells were washed and incubated with the appropriate secondary Alexa Fluor‐conjugated antibody (dilution 1:500; Thermo Fisher Scientific) at room temperature for 2 hr. After washes, cells were permeabilized for 10 min with PBS + 0.25% Triton X‐100, washed, and incubated overnight at 4ºC with MAP2 or MAP2 and PSD95 primary antibodies. Cells were washed and incubated with the appropriate secondary Alexa Fluor‐conjugated antibody (dilution 1:500; Thermo Fisher Scientific) at room temperature for 2h. After washes, cells were mounted with Fluoromount‐G (SouthernBiotech). Pictures were taken under an EVOSfl (AMG) fluorescence microscope. Fluorescence intensity was quantified on straightened 50µm dendrite sections using ImageJ v1.51j8.

### 4.8 Electrochemiluminescence sandwich immunoassay

Aβ peptides levels were quantified using the MSD multispot Aβ 6E10 assay kit (cat K15200E Meso Scale Discovery), following the manufacturer's instructions with the following modification: Undiluted S2 samples and standards were loaded onto the plate and incubated overnight at 4ºC before reading. Readings were normalized to total protein content determined using a commercial Bradford assay (Bio‐Rad).

### 4.9 Immunohistochemistry

Five‐µm coronal sections were deparaffinized with xylenes and rehydrated in graded ethanol series. Sections were microwaved with citrate buffer for 20 min and pretreated with 90% formic acid for 4 min and with 3% H_2_0_2_/3% methanol in Tris‐buffered saline (TBS) for 30 min to block endogenous peroxide activity. After TBS washes, sections were incubated first in TBS with 0.1% Triton X‐100 (TBST) for 15 min, and then in TBST with 3% bovine serum albumin (BSA, Sigma‐Aldrich) for 30 min. Following, sections were blocked 1 hr in TBST with 3% BSA and 2% horse serum (Vector Labs.). Sections were incubated overnight with primary antibody in TBS + 3% BSA + 2% horse serum at 4ºC. After washes, sections were incubated with biotinylated secondary antibody (1:500, Vector Labs.) in TBS + 3% BSA + 2% horse serum at room temperature for 1 hr, followed by Vector ABC Kit and DAB reagents (Vector Labs.). After, sections were dehydrated, cleared, and coverslipped with DPX (BDH) mounting medium. The specificity of the immune reactions was controlled by omitting the primary antibody. Aβ plaque burden and HT7 positive signal were analyzed in the CA1 region of the hippocampus. Sections were observed under an Axioskop (Zeiss) microscope and acquired with an AxioCam HRc (Zeiss) digital camera. The camera settings were adjusted at the start of the experiment and maintained for uniformity. Digital images (4 sections per animal, *n* = 4–6 per group) were analyzed using Image J software. Positive signal was identified by threshold level which was maintained constant throughout the experiments.

### 4.10 Flow cytometry

To obtain fresh crude synaptosome P2 fractions, hippocampi were rapidly dissected from a single mouse and homogenized in 320 mM sucrose (1.5 ml) containing HEPES [10 mM] and protease/phosphatase inhibitors cocktail (Pierce), pH 7.4, 4°C. Homogenization consisted of 6–8 manual strokes in a glass–teflon grinder, clearance (between plunger and glass): 0.15–0.25 mm. The homogenate was centrifuged at 1,200 × *g* for 10 min. 4°C; supernatant (S1, containing mitochondria and synaptosomes) was transferred into microfuge tubes and centrifuged at 12,000 × *g* for 20 min. Supernatant (S2) were carefully removed, and the pellet (P2, corresponding to the crude synaptosome fraction) was resuspended by gently pipetting up and down (10–20 times) in PBS. Samples were protected from light, maintained at 4°C, and run on a flow cytometer (Novocyte, ACEA Bioscience, Inc) immediately. FSC, SSC, and BL1‐fluorescence [530 ± 30 nm] signals were collected using log amplification. FSC‐SSC plots were used to select particles matching the size of synaptosomes (~1.0 μm) using calibrated beads (Polysciences, Inc.), as previously described (Prieto et al., 2017). Settings for fluorescence amplification on BL1 detector (for GFP) were based on the emission of size‐based gated synaptosomes of an uninjected animal. Total 20,000 events were collected and analyzed for each sample, event rate: approximately 800/sec. Analysis was performed using the FlowJo software (LLC).

### 4.11 Statistical analysis

Comparisons between multiple groups used one‐way analysis of variance (ANOVA) followed by Fisher's post hoc tests. Comparisons between two groups used Student's *t* test. *p*‐value ≤ .05 was considered significant. All values represent the mean + *SEM*.

## CONFLICT OF INTEREST

The authors declared no potential conflict of interests with respect to this article.

## AUTHOR CONTRIBUTIONS

CRO and MK conceived and designed the experiments. CRO, GAP, ACM, SF, LTE, and DBV performed the experiments. CRO, GAP, LTE, and DBV analyzed the data. CRO, GAP, ACM, SF, LTE, FML, DBV, CWC, and MK contributed to the writing of the manuscript.
