# Supplementary figures and images for: miR‐181a negatively modulates synaptic plasticity in hippocampal cultures and its inhibition rescues memory deficits in a mouse model of Alzheimer’s disease
Source: Aging Cell. 2020 Feb 22;19(3):e13118. doi: 10.1111/acel.13118 (PMC7059142; doi:10.1111/acel.13118)

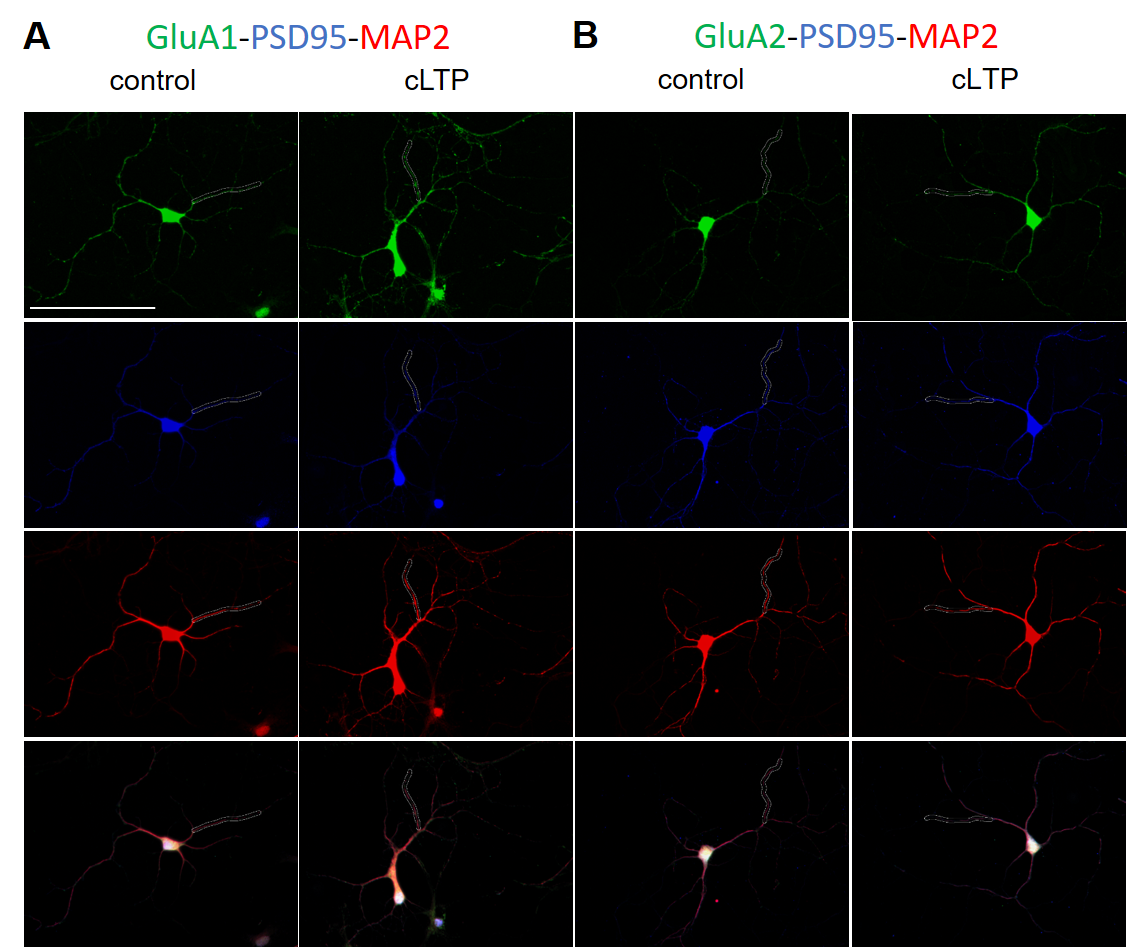

Supplement: Supplementary file 1 [file ACEL-19-e13118-s001.png]

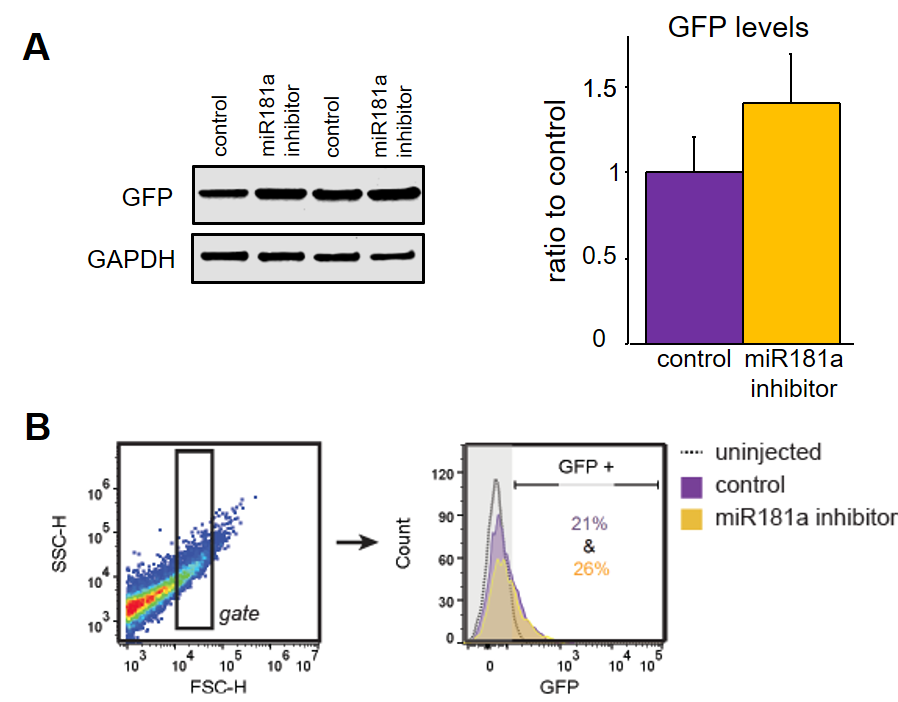

Supplement: Supplementary file 2 [file ACEL-19-e13118-s002.png]

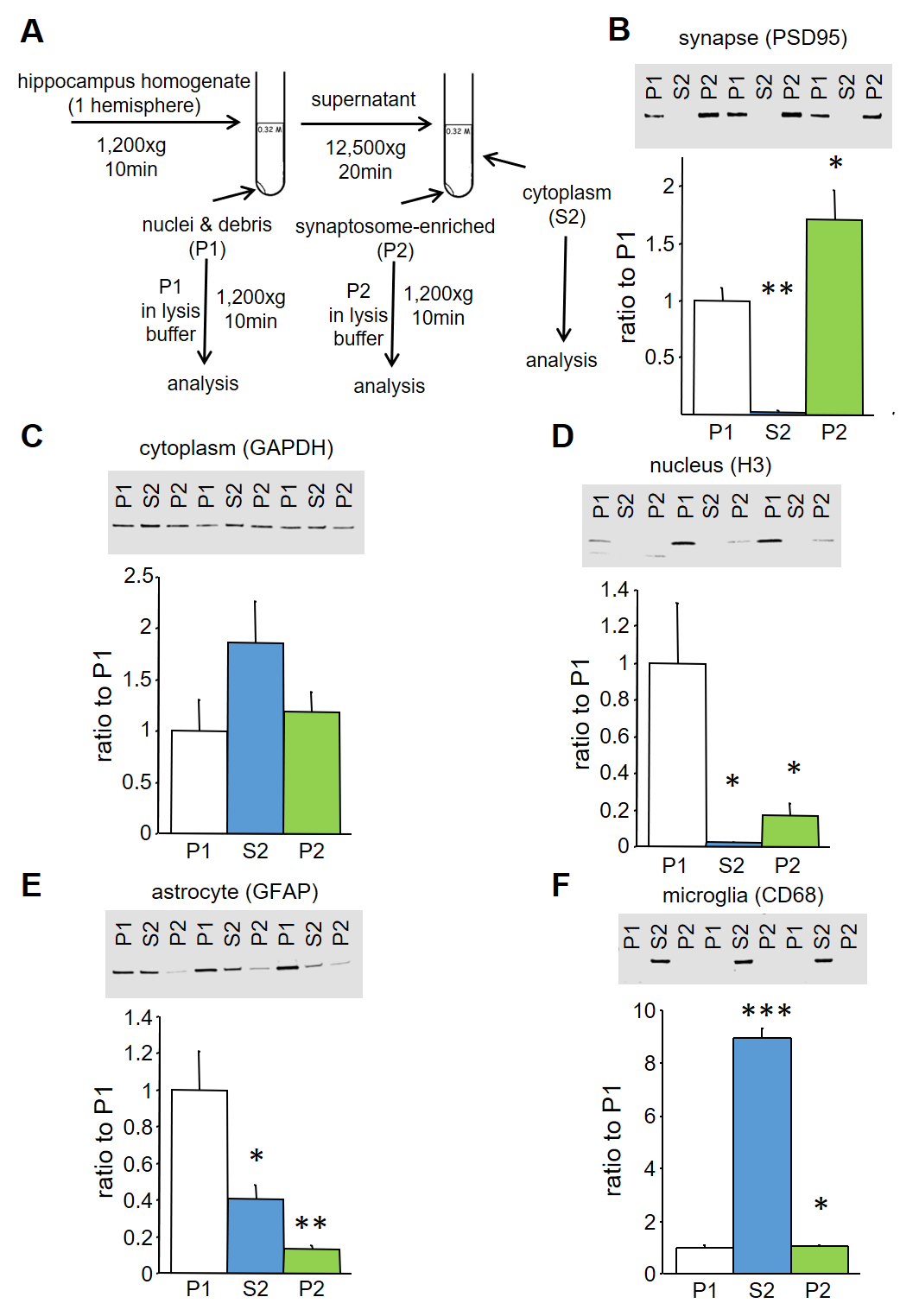

Supplement: Supplementary file 3 [file ACEL-19-e13118-s003.png]

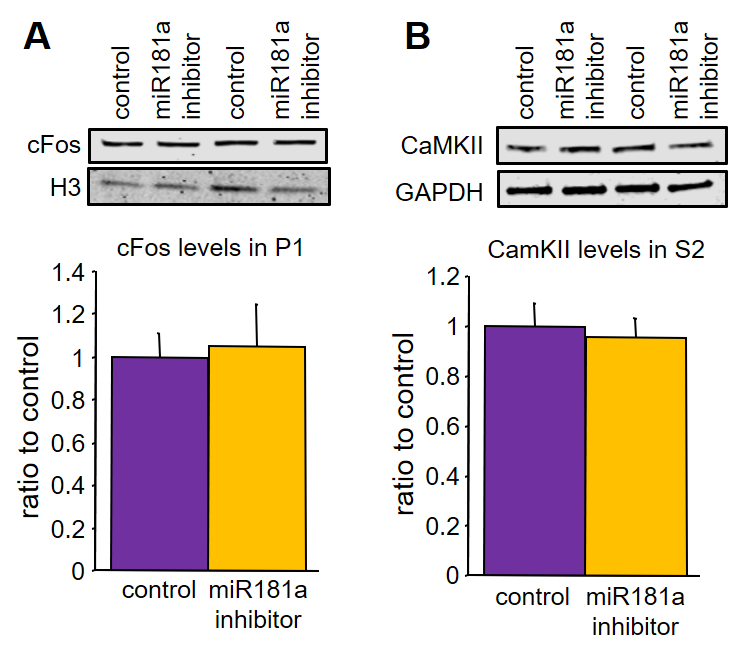

Supplement: Supplementary file 4 [file ACEL-19-e13118-s004.png]
